# Prevalence of osteoporosis and associated factors among people aged 50 years and older in the Madhesh province of Nepal: a community-based cross-sectional study

**DOI:** 10.1186/s41043-024-00591-7

**Published:** 2024-07-04

**Authors:** Narendra Kumar Chaudhary, Dev Ram Sunuwar, Manish Raj Sapkota, Suman Pant, Mary Pradhan, Kailash Kumar Bhandari

**Affiliations:** 1 Department of Radiology, Nepal Orthopaedic Hospital, Kathmandu, Nepal; 2 Department of Nutritional Science, School of Public Health, University of Michigan, Ann Arbor, USA; 3 Department of Biology, Texas A&M University, College Station, USA; 4 Nepal Health Research Council, Kathmandu, Nepal; 5 Kantipur Academy of Health Science, Kathmandu, Nepal; 6 Department of Orthopaedics, Nepal Orthopaedic Hospital, Kathmandu, Nepal

**Keywords:** Bone mineral density (BMD), Osteoporosis, Osteoporosis self-assessment tools for Asians (OSTA)

## Abstract

**Background:**

The high prevalence of osteoporosis has increased the economic burden on the health system globally. The burden of osteoporosis and its associated factors have not been adequately assessed in community settings in the Nepalese context thus far. Therefore, this study aimed to assess the prevalence of osteoporosis and its associated factors, lifestyle behaviors, and dietary calcium intake.

**Methods:**

A community-based cross-sectional study was conducted among 395 people aged 50 years and older in the *Madhesh* Province of Nepal between July 2022 and August 2023. The Osteoporosis Self-assessment Tools for Asians (OSTA) index was used to measure osteoporosis. A structured questionnaire was used to collect sociodemographic information, anthropometric data, lifestyle behavior, daily dietary calcium intake, and frequency of calcium-rich food consumption. A food frequency questionnaire and 24-hour recall methods were used to assess dietary intake. The chi-square test, binary logistic regression and Mann‒Whitney U test were applied to measure the association between predictors and the outcome of interest.

**Results:**

The prevalence of no risk, moderate risk and high risk of osteoporosis were 38.7%, 39%, and 22.3% respectively. The risk of osteoporosis was higher in females (aOR = 5.18, CI: 2.10-12.75, *p* < 0.001) and increased risk with advancing age (aOR = 32.49, CI: 14.02–75.28, *p* < 0.001). Similarly, underweight was associated with increased odds of having osteoporosis (aOR = 13.42, CI = 4.58–39.30, *p* < 0.001). The incidence of osteoporosis was strongly associated with daily calcium intake of 225 mg (100, 386).

**Conclusion:**

This study revealed a high prevalence of osteoporosis among people aged 50 years and older due to the combined effect of being underweight and having inadequate calcium intake. Nutritional counselling services encourage people to consume sufficient calcium-rich food and adopt an appropriate lifestyle behaviours to maintain healthy body weight so that osteoporosis and osteoporotic fractures could be prevented. Further research can explore the impact of socioeconomic status and medical comorbidities on a large scale.

## Introduction

Osteoporosis, known as a silent disease in the 21st century [1], is a major public health concern worldwide [2, 3] because it generally does not manifest until an osteoporotic fracture occurs [4]. It impacts people from all backgrounds of life [5] and therefore has been a clinical issue [6]. Osteoporosis is a skeletal disorder characterized by the loss of bone mass and microarchitectural deterioration of bone tissue, which leads to decreased bone tension and strength, increasing the risk of fragility fractures [7, 8]. The most common sites of fracture are the femoral neck, hip, lumbar and thoracic spine, and distal wrist [9]. Osteoporotic fracture worsens the quality of life and quality-adjusted life annually [10], limiting autonomy, increasing disability and decreasing life expectancy [11]. Osteoporotic fracture is predicted to increase by 20% by 2035 and dramatically up to 135% by 2040 annually in China, where the proportion of the population 50 years and older is expected to double [12]. Moreover, these trend increase the medical costs that impose a significant economic burden on the national health system worldwide [13]. Adami et al. 2022, report that the treatment cost of osteoporosis in 2007 will increase by 50% by 2025, which holds true to date [12].

Osteoporosis is a multifactorial disorder caused by the interaction between environmental factors and genes that exert modest effects on bone metabolism [14]. Age, sex, ethnicity, race, heredity, lifestyle and dietary habits are the contributing factors to the occurrence of osteoporosis [15]. In Vietnam and Thailand, the incidence of bone loss is alarming in people after 70 years of age among seven Southeast Asian countries [16]. Rychter et al., 2021 report that different lifestyle behaviors, such as reduced physical activity, smoking and alcohol consumption, lead to a risk of osteoporotic fracture [17]. Chronic use of glucocorticoids, lifestyle conditions, habits, and major depression are predisposing factors for osteoporosis [18].

The global prevalence of osteoporosis is 18.3%, of which Asia has the highest prevalence (24.3%) among Europe (16.7%) and the USA (11.5%) [5]. In Nepal, little is known about the prevalence of osteoporosis and its causal factors in clinical setting with bone densitometry method. Bone densitometry is the most preferred test for assessing the density or thickness of bone for evaluating bone strength and the risk of fractures [19]. The prevalence of osteoporosis is 37.3% in adults aged 50 years and older according to the Dual energy X-ray absorptiometry (DXA) method [20], which is considered the gold standard densitometry method for confirming osteoporosis [21]. A bone mineral density (BMD) value with a T score ≤ -2.5 SD at either the femoral neck or lumbosacral spine is considered the cut-off value for the evaluation of osteoporosis [22]. Another study done with the quantitative ultrasound (QUS) densitometry method indicates the prevalence of osteoporosis as 31.57% in people aged 50 to 59 years and 60.5% in people aged 60 years and older [23]. Similarly, Dhakal et al. 2024 reveal the prevalence of osteoporosis is 51.7% in elder people (≥ 60 years) with QUS [24]. QUS is a low cost and readily available alternative to DXA measurement of the BMD to evaluate the osteoporotic fracture risk assessment [25]. However, these imaging modalities cannot be applied at most community levels due to the high cost, lack of wide availability and unavailability of licenced radiographic technicians to perform the scan [26]. Bui et al. 2022 reported that community-based screening tools, such as osteoporosis self-assessment tools for Asians (OSTA), had greater predictive value (80.0%) for the DXA of femoral necks among postmenopausal Vietnamese women [27]. A study also revealed that an OSTA ≤ − 1 has a sensitivity of 81% and specificity of 66% compared to DXA [28]. In Singapore, the prevalence of osteoporosis at the community level among people aged > 60 years is 52%, as determined by the use of the OSTA [28]. Approximately 17.9% of the men aged 50 years and above were diagnosed as osteoporosis with OSTA in North India [29]. However, no studies have been conducted in Nepal to determine the prevalence of osteoporosis at the community level. Therefore, this study aimed to determine the risk of osteoporosis and its association with food consumption, especially the daily dietary calcium consumption and lifestyle behaviour in a community setting.

## Materials and methods

### Study design and setting

This study followed a community-based cross-sectional study in the *Saptari* district of *Madhesh* Province, Nepal, which started in July 2022 to August 2023. The *Madhesh* Province lies between 84^0^27′ and 86^0^54’30” east longitude and 26^0^23’38” and 27^0^28’17” north latitude and occupies approximately 6.56% of the total area of the country [30]. This province has a dense population; according to the 2021 National Population Census, there are 6,126,288 (20.98% of total Nepalese) people in *Madhesh* Province, and among them, 713,203 people are in the *Saptari* district (523.3/km^2^ population density) [31]. There are 116 ethnic groups in *Madhesh* Province, of which the largest ethnic group is *Yadav* (14.78%), followed by 11.58% *Muslim*, 5.26% *Tharu*, 5.09% *Teli*, and 4.56% *Koiri/Kushwaha*. Similarly, on the basis of religion, the majority of the population is Hindu (82.8%), followed by Islam (9%), Buddhism (4.4%), Kirat (3%), and Christianity (1.3%). According to the 2021 census, 5,380,242 (71.97%) of the total population of the province reside in urban areas [32]. In the *Saptari* district, there are also diverse ethnic and multi-religious people with varying maternal tongue resides. According to the 2011 report, 35% of the population were *Madhesi*, 24% were *Janajati*, 23% were *Dalit*, 8% were *Muslims*, 4% were *Brahmin*, 2% were *Chhetri*, and there were smaller shares of other ethnic groups in the *Saptari* district. On the basis of religion, 85% were Hindu, 9% were Islamic and 5% were Buddhist [33]. This finding matches the census of *Madhesh*.

For this study, samples from individuals aged 50 years and above who had an active life and good cognitive status were chosen because bone mass remains stable until approximately 50 years of age, after which the imbalance of the bone remodelling process appears to lead to osteoporosis [34]. The respondents were informed about the purpose of this study, and only those who provided written informed consent were included in the study. With regard to illiterate participants, their thumb stamps were considered to indicate that they signed consent forms in the presence of a literate witness whom they knew well. However, individuals who were receiving calcium or vitamin D supplementation, who were recently fractured within six- months or who underwent any surgery in the femur or spine were excluded from the study.

### Sample size and sampling strategy

The sample size was determined using a single proportional formula (n = Z^2^pq/d^2)^, and the prevalence rate of osteoporosis was 37.3% according to a previous study [20]. In the formula, Z = standard normal deviation and equals 1.96 at the 95% significance level; p is the prevalence of osteoporosis, which was set at 0.37; q = 1-p; and the margin error (d) was set at 5%. Hence, by adding a 10% non-response rate, the sample size was 395.

**Fig. 1:**
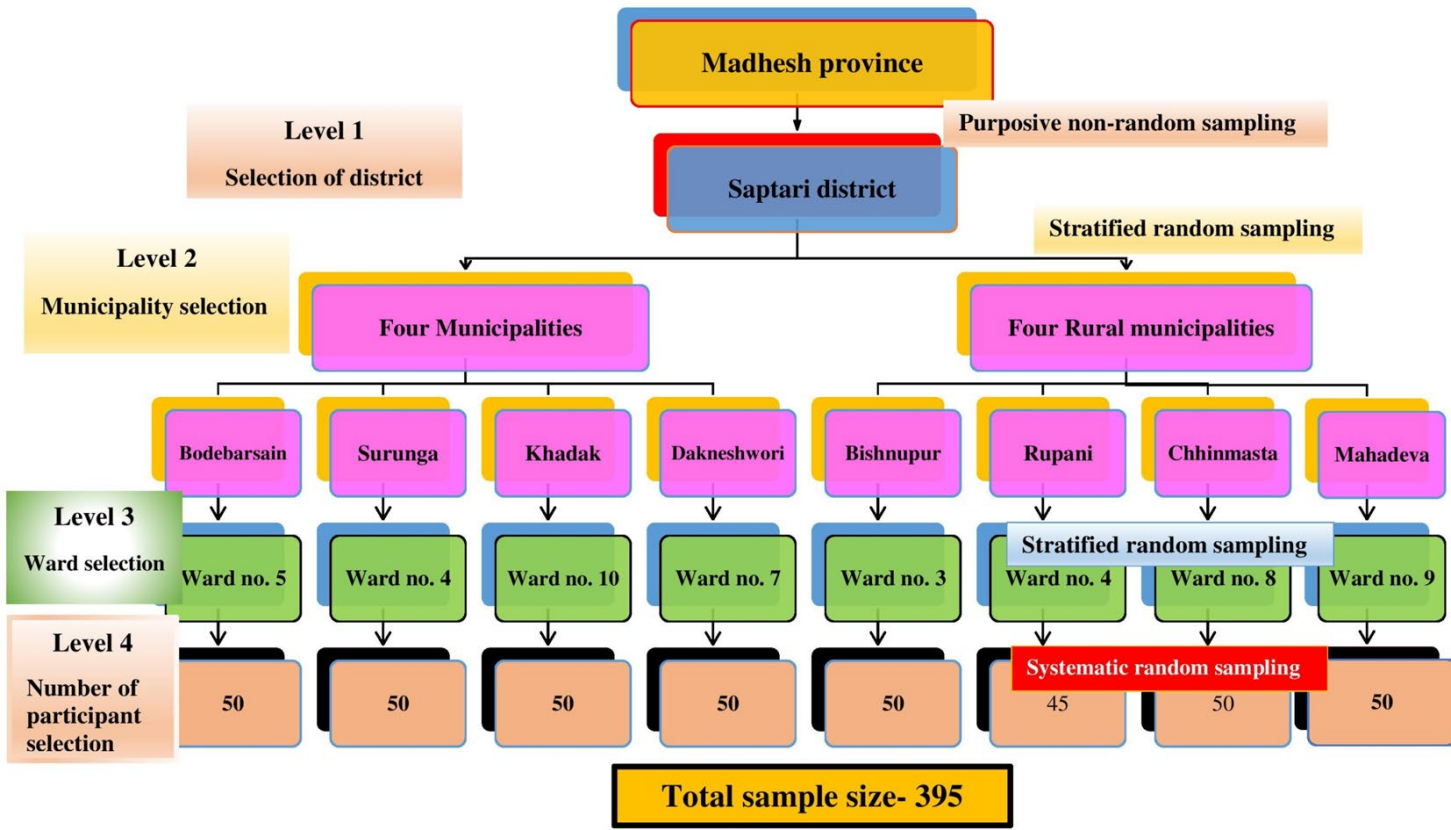
Multilevel mixed method sampling design

This study followed the multi-level mixed methods sampling design which involves the combination of different sampling techniques either purposive sampling or random sampling at various levels to collect the comprehensive data for the evaluation of desired outcome. These designs of sampling are mentioned in different literatures [35–37]. In the *Madhesh* province of Nepal, there are eight districts; *Parsa, Bara, Rauthat, Sarlahi, Mahaotari, Dhanusha, Siraha* and *Saptari*. *Saptari* district was chosen through purposive non-random sampling method as the research site in the first level of sampling design. In the *Saptari* district, there are nine urban municipalities (simply called only municipality); *Dakneshwori, Rajbiraj, Bodebarsain, Hanumannagar Yoginimai, Kanchanrup, Saptakoshi, Surunga, Shambhunath* and *Khadak*, and nine rural municipalities; *Bishnupur, Rajgad, Chhinmasta, Tilathi Koiladi, Mahadeva, Agnisaira Krishna Savaran, Tirhut, Balan-bihul* and *Rupani*. Municipalities are the administrative division of province on the basis of population and geographical area in Nepal. These urban and rural municipalities were considered as the different strata for this research. In the second level of sampling design, four urban and four rural municipalities were selected through stratified random sampling method. This selection was designed to ensure a balanced representation of both urban and rural areas within the sample. There are total 164 wards in *Saptari* district ranging from 9 to 11 in number in each municipality. Ward is the smallest unit of local government on the hierarchy of the administrative division of Nepal and it falls under the urban and rural municipality. These wards were also considered as the strata for the sampling design and therefore at the third level of sampling design, one ward from each municipality was selected through stratified random sampling method. Subsequently, 50 households were chosen from each designated ward except from the ward from *Rupani* rural municipality as it is the smallest municipality in the district. A list of households was compiled from each ward, and systematic random sampling was employed to select the households. From each selected household, one participant having eligible criteria was chosen. If more than one participants were eligible to participate from the designated household, the lottery method was applied to select only one participant. The eligible participant of another adjacent household was selected if there was the absence of an eligible participant in any household (Fig. 1).

### Data collection technique and study variables

The principal investigator collected the data using a structured questionnaire that we adapted from a previously published study [20] and modified the questionnaire based on our context. Subsequently, we conducted a pilot study with 10% of the total sample size in the neighbouring municipality to assess the consistency, clarity, and accuracy of the questionnaire. Face-to-face interviews were conducted at the participants’ residences for at least 30 min, and the questionnaire was also completed in front of the participants. The questionnaire consisted of different components: sociodemographic information (age, sex, education, religion, ethnicity and occupation), anthropometric information (height and weight), food consumption throughout the day prior to the interview day, frequency of consumption of calcium-rich food for one week and lifestyle behaviour-related factors (smoking habit and alcohol intake). For the reliability of the study, the height and weight of the participants were measured three times, and the mean values were recorded. The questionnaires were checked daily for completeness, consistency and clarity, as mentioned earlier. Proper probing was performed to minimize interview bias, and sufficient time was allocated for the interviews.

All the tools were originally developed in the English language, translated into the Nepali language and subsequently back-translated to English to ensure their validity (and reliability). Piloting was performed on 10% of the total sample to check the language and clarity of the questions at the study site after ethical approval was obtained. Sufficient time was provided to understand the aim and rationality of the study and to remember the food consumed last day. If the participants were illiterate, assistance was obtained from a literate family member or other person on whom the participants believed. After piloting, all the ambiguous, misleading and wrongly interpreted questions were omitted, and the questionnaire was revised in accordance with the findings of the piloting.

### Predictor variables

#### Sociodemographic information

Sociodemographic information such as age, sex, educational level, ethnicity, religion status and occupation was recorded. For age, two categories, 50 to 59 years and 60 years and above were made for analysis. Similarly, gender was categorized as male or female. Educational status was categorized as no formal education for illiterate participants who were never involved in formal schooling, primary education who could simply read and write, and an School Leaving Certificate (SLC) or above who had passed the annual examination administered at Grade 10 students within the Nepalese secondary school system, which is considered the iron gate of education in Nepal [38]. Even though there are multiple ethnic groups in Nepal, the ethnicities were categorized as *Tharus* (one of the oldest and largest indigenous groups of *Terai*) [39], *Dalit* (those ethnic minorities who fall among the disadvantaged groups according to several socioeconomic indicators) [40] and others (*Brahamin/Chhetri)* who are considered to have greater caste and enjoy the greatest social, economic and political access) [41] for ease of analysis. Similarly religion was categorized as Hindu or non-Hindu, with 80% of the total Nepalese population following Hindu religion and the rest following other religions [42]. Since more than 80% of the total Nepalese population is dependent on agriculture [43], the participants were categorized as farming or non-farming for the convenience of analysis in the occupation variable.

#### Anthropometric measurements

Anthropometric measurements, such as height and weight, were taken according to standardized procedures. For this study, height was measured while the participants were in the standing position to the nearest 0.1 cm with a Seca 213 portable stadiometer. Weight was measured to the nearest 0.1 kg using an Omron digital weighing machine. Body mass index (BMI, kg/m^2^) was calculated and categorized using the World Health Organization (WHO) cut-off points for BMI for Asians [44].

#### Lifestyle behaviour-related factors

Alcohol consumption and smoking habits were considered as lifestyle factors. For ease of analysis, smoking habit and alcohol consumption were categorized as either ‘Yes’ or ‘No’. To mark ‘No’, the participants were required to neither have smoked nor consumed alcohol for the past six months; otherwise, they were asked to mark ‘Yes’.

#### Dietary calcium intake

Daily dietary calcium intake was assessed by assessing the participants’ 24-hour recall. Participants were asked to name the foods and beverages consumed and their quantities during the past 24 h (from midnight to midnight of the previous day). For ease, the household measurement of a 130 ml cup for one serving was displayed. Dietary daily calcium intake was calculated manually with the help of the 2012 food composition table in Nepal [45]. To make this easier, calcium-rich foods were listed, and their calcium content was measured per household. Similarly, the sufficiency of calcium-rich food consumption was explored through a one-week food frequency questionnaire. We identified and listed the high-calcium-rich foods that are mostly consumed in the *Madhesh* province. There are different ethnic indigenous people living in the *Terai* region of *Madhesh* Province. Most of them consume fresh water snails, known as *GHONGHIs* in the local language (scientific name: *Bellamya bengalensis*), which are especially popular in *Tharu* ethnic groups. It contains very high amounts of calcium and phosphorus and is pivotal for healthy bone. According to the 2012 food composition table of Nepal, 100 g of edible snail contains 1321 milligrams of calcium and 147 milligrams of phosphorus, which are very high and generally not found in other foods naturally [45].

#### Outcome variables

The prevalence of osteoporosis was predicted by the Osteoporosis Self-assessment tool for Asians (OSTA). The OSTA score was calculated by subtracting age in years from weight in kilograms and multiplying by 0.2, which is based on several studies [46–49]. Participants were classified into three categories: low risk, intermediate risk and high risk. The results are presented on the basis of sex; for men, low risk >-1, intermediate risk − 1 to -6 and high risk <-6. For women, low risk >-1, intermediate risk − 1 to -4 and high risk <-4 [26].

#### Data management and analysis

The data were entered into EpiData version 3.2 and analysed on the basis of the intention-to treat (ITT) approach using Stata/MP version 14.1 (Stata Corp LP, College Station, Texas). The incidence of osteoporosis was estimated through the use of descriptive statistical tools and the OSTA. The chi-square test was applied to determine the associations of the sociodemographic profile, lifestyle behavior and dietary calcium intake with osteoporosis. The Mann‒Whitney U test was applied to compare osteoporosis prevalence and the median daily dietary calcium intake score. All associations with probability values less than 0.05 (*p* < 0.05) were considered to indicate statistical significance. Binary logistic regression analysis was applied for multivariate analysis, and a p value less than 0.05 was considered to indicate statistical significance (*p* < 0.05).

#### Ethical considerations

This study was conducted only after obtaining ethical approval from the Ethical Review Board (ERB) of the Nepal Health Research Council (NHRC), Kathmandu, Nepal (Reference number: 2909). Similarly, permission was also obtained from the respective municipalities before the participants were approached at the household level.

## Results

### Sociodemographic characteristics

Table 1 shows the characteristics of the participants, including sociodemographic characteristics, anthropometric data, bone status, dietary calcium consumption and behaviour-related factors.

There were 395 participants, among whom 219 (55.4%) were male and 176 (44.6%) were female. Similarly, 185 (46.8%) of the participants were aged 50 to 59 years, and 210 (53.2%) were aged 60 years and older. The mean age of the participants was 61.78 years, and the standard deviation (SD) was 9.44 years. Likewise, 256 (64.8%) had no formal education, 92 (23.3%) had primary education, and only 47 (11.9%) had a history of SLC or above. On the basis of ethnicity, there were 221 (55.9%) *Tharu*, 142 (35.9%) *Dalit* and 32 (8.1%) others. Most of the participants, 368 (93.2%), were religious Hindu followers, while 27 (6.8%) were religious non-Hindu followers. Agriculture was the occupation for most of the participants, accounting for 388 (98.2%) (Table 1).

### Anthropometric measurements

The mean height and weight of the participants were 124 ± 28.52 cm and 27 ± 12.26 kg, respectively. On the basis of height and weight, BMI was calculated and found to be 21.17 ± 3.76 kg/m^2^ as the mean ± SD BMI. When BMI was categorized, 230 (58.2%) had a normal BMI, 102 (25.8%) were underweight, and 63 (15.9%) were overweight/obese (Table 1).

### Prevalence of osteoporosis

According to the OSTA, 153 (38.7%) had no risk of osteoporosis, 154 (39.0%) had a moderate risk of osteoporosis, and 88 (22.3%) had a severe risk of osteoporosis. The mean ± SD of the OSTA was − 1.93 ± 3.67. The mean ± SD OSTA was − 0.99 ± 3.77 for males and − 3.09 ± 3.17 for females. The prevalence of osteoporosis was 74.3% among females, while that among males was 50.9% (Tables 1 and 2).

### Dietary calcium intake

The daily dietary calcium intake was found to be 360.87 ± 362.39 mg. Similarly, on the basis of the one-week food frequency questionnaire, it was found that most of the participants (71.7%) did not consume calcium-rich food daily (Table 1).

### Behaviour-related factors

Among the 395 participants, more than half (64.5%) had no smoking habit. Similarly, more than two-thirds of the participants (81.1%) did not consume alcohol (Table 1).

**Table 1:** Socio-demographic characteristics of the participants (*n* = 395)

Variables	Frequency (%)
**Socio demographic characteristics**	
**Gender**	
Male	219 (55.4)
Female	176 (44.6)
**Age**	
50–59 years	185 (46.8)
60 and above years	210 (53.2)
**Age in years, mean ± SD**	**61.78 ± 9.44**
**Education**	
No formal education	256 (64.8)
Primary education	92 (23.3)
SLC or above	47 (11.9)
**Ethnicity**	
*Tharu*	221 (55.9%)
*Dalit*	142 (35.9)
Others	32 (8.1)
**Religion**	
Hindu	368 (93.2)
Non-Hindu	27 (6.8)
**Occupation**	
Farming	388 (98.2)
Others	7 (1.8)
**Anthropometric measurement**	
Height in cm, mean ± SD	124 ± 28.52
Weight in kg, mean ± SD	27 ± 12.26
**BMI (mean ± SD)**	**21.17 ± 3.76**
BMI categories	
Normal	230 (58.2)
Underweight	102 (25.8)
Overweight/obese	63 (15.9)
**Osteoporosis status**	
**OSTA score, mean ± SD**	**-1.93 ± 3.671**
**OSTA classification**	
No Risk	153 (38.7)
Moderate Risk	154 (39.0)
High Risk	88 (22.3)
**OSTA value, mean** ± **SD**	
Male	-0.99 (3.77)
Female	-3.09 (3.17)
**Daily Calcium intake in mg, mean ± SD**	**360.87 ± 362.39**
**Calcium intake**	
Sufficient	112 (28.3)
Not sufficient	283 (71.7)
**Behaviour related factors**	
**Smoking**	
No	255 (64.5)
Yes	140 (35.5)
**Alcohol**	
No	320 (81.1)
Yes	75 (18.9)

### Associations of osteoporosis with different variables

Regarding sociodemographic variables, females had almost 3 times greater odds of having osteoporosis than males did (cOR = 2.83; 95% CI = 1.84–4.35, p value < 0.001). Similarly, people aged *≥* 60 years had an approximately 18-fold greater chance of having osteoporosis than people aged 50–59 years (cOR = 17.85; 95% CI = 10.55–30.27, p value < 0.001). Regarding literacy, participants with no formal education had a 10-fold greater chance of suffering from osteoporosis (cOR = 10.02; 95% CI = 4.81–20.86, p value < 0.001). With respect to the *Tharu* ethnic group, the *Dalit* ethnic group had a 2-fold greater chance of developing osteoporosis (cOR = 2.30; 95% CI = 1.45–3.56, p value < 0.001). Neither religion nor occupation was associated with osteoporosis incidence. Regarding BMI as an anthropometric indicator, participants who were underweight had 10 times greater odds of having osteoporosis than participants with a normal BMI (cOR = 10.28; 95% CI = 4.32–24.45, p value < 0.001). Similarly, from the diet perspective, the frequency of intake of calcium-rich food was associated with osteoporosis (cOR = 2.01; 95% CI = 1.29–3.14, p value = 0.002). Among the behaviour-related factors, smoking was not associated, but alcohol consumption had a protective effect on osteoporosis (cOR = 0.27; 95% CI = 0.16–0.46, p value < 0.001) (Table 2).

### Multivariate analysis

According to the adjusted model, females had 5 times greater odds of having osteoporosis than males did (aOR = 5.18; 95% CI = 2.10-12.75, p value < 0.001). Similarly, participants aged *≥* 60 years had a greater chance of having osteoporosis than people aged 50–59 years (aOR = 32.49; 95% CI = 14.02–75.28, p value < 0.001). However, neither literacy status nor ethnicity was associated with osteoporosis (aOR = 1.79; 95% CI = 0.57–5.54, p value = 0.311 and aOR = 1.86; 95% CI = 0.86–4.02, p value = 0.112, respectively). Regarding BMI, underweight individuals had 13 times greater odds of having osteoporosis than did individuals with a normal BMI and overweight/obese individuals (aOR 13.42; 95% CI = 4.58–39.30, p value < 0.001). The frequency of intake of calcium-rich food did not show any association with osteoporosis (aOR = 1.72; 95% CI = 0.85–3.55, p value = 0.127). Alcohol consumption had no association with the occurrence of osteoporosis (aOR = 1.02; 95% CI = 0.37–2.80, p value = 0.955) (Table 2).

**Fig. 2:**
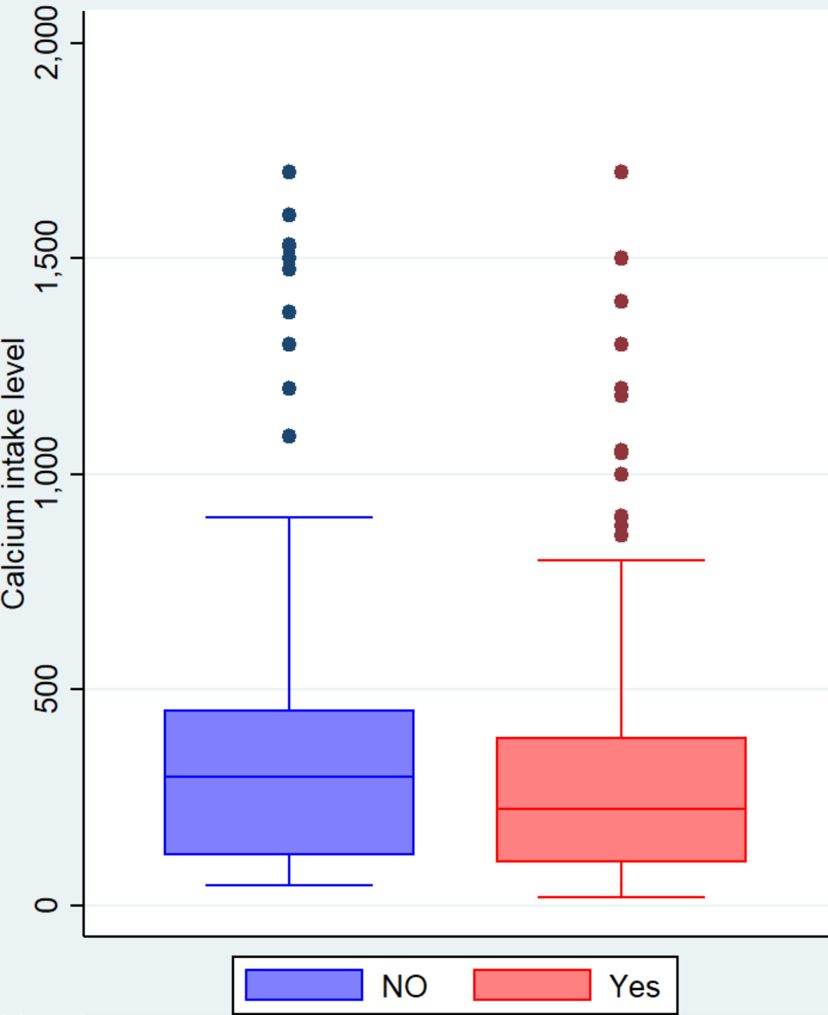
Box and whisker plot of daily dietary calcium consumption

The findings showed a strong association between calcium intake and osteoporosis, with a median calcium consumption of 300 mg and an interquartile range (IQR) of 115 to 450 among the participants with no osteoporosis, while the median calcium intake was 225 mg, with an IQR of 100 to 386, among those participants with osteoporosis (Fig. 2; Table 3).

**Table 2:** Associations of osteoporosis risk with sociodemographic, anthropometric and lifestyle behaviors (*n* = 395)

Variables	Risk of osteoporosis		Bivariate analysis		Multivariable analysis	
	No Number (%)	Yes Number (%)	cOR (95% CI)	*P* value^1^	aOR (95% CI)	*P* value^2^
**Sociodemographic characteristics**						
**Gender**						
Male	108 (49.3)	111 (50.9)	Ref		Ref	
Female	45 (25.7)	131 (74.3)	2.83 (1.84-4.35)	<0.001*	5.18(2.10-12.75)	<0.001*
**Age**						
50–59	129 (69.7)	56 (30.3)	Ref		Ref	
60 and above	24 (11.4)	186 (88.7)	17.85 (10.55-30.27)	<0.001*	32.49 (14.02-75.28)	<0.001*
**Education**						
No formal education	63 (24.6)	193 (75.4)	10.02 (4.81-20.86)	<0.001*	1.79 (0.57-5.54)	0.311 0.301
Primary education	54 (58.8)	38 (41.2)	2.30 (1.04-5.08)	0.039*	1.26 (0.40-3.90)	
SLC or above	36 (76.6)	11 (23.4)	Ref		Ref	
**Ethnicity**						
*Tharu*	97 (43.9)	124 (56.1)	Ref		Ref	
*Dalit*	36 (25.4)	106 (74.6)	2.30 (1.45-3.56)	<0.001*	1.86 (0.86-4.02)	0.112
Others	20 (62.5)	12 (37.5)	0.46 (0.21-1.00)	0.052	0.06 (0.003-10.35)	0.301
**Religion**						
Hindu	138 (37.5)	230 (62.5)	2.08 (0.94-4.58)	0.068		
Non-Hindu	15 (55.5)	12 (44.5)	Ref			
**Occupation**						
Farming	149 (38.4)	239 (61.6)	2.13 (0.47-9.68)	0.324		
Others	4 (57.1)	3 (42.8)	Ref			
**Anthropometric factors**						
BMI categories						
Normal	90 (39.1)	140 (60.9)	Ref		Ref	
Underweight	6 (5.8)	96 (94.1)	10.28 (4.32-24.45)	<0.001*	13.42 (4.58-39.30)	<0.001*
Overweight/obese	57 (90.5)	6 (9.5)	0.06 (0.02-0.16)	<0.001*	0.04 (0.01-0.15)	<0.001*
**Calcium intake**						
Sufficient	57 (50.9)	55 (49.1)	Ref		Ref	
Not sufficient	96 (33.9)	187 (66.1)	2.01 (1.29-3.14)	0.002*	1.72 (0.85-3.55)	0.127
**Behaviour related factors**						
**Smoking**						
No	101 (39.6)	154 (60.4)	Ref			
Yes	52 (37.1)	88 (62.9)	1.10 (0.72-1.69)	0.631		
**Alcohol**						
No	105 (32.8)	215 (67.2)	Ref		Ref	
Yes	48 (64.0)	27 (36.0)	0.27 (0.16-0.46)	<0.001*	1.02 (0.37-2.80)	0.955

^1^Unadjusted model: cOR: Crude Odds Ratio; ^2^Adjusted model: aOR: Adjusted odds ratio; In the multivariable analysis, only significant variables from the bivariate model were adjusted

**Table 3:** Association of daily dietary calcium intake with osteoporosis

Osteoporosis	Daily dietary calcium intake		
	Frequency	Median (IQR)^a^	*P* value
No	153	300 (115, 450)	<0.001*
Yes	242	225 (100, 386)	

^a^ Mann–Whitney U test

## Discussion

This study is the first of its kind to assess the factors associated with osteoporosis in a community setting in Nepal. This study showed the prevalence of osteoporosis among approximately one fourth (22.3%) people aged 50 years and older. The prevalence was found to be increased in higher age group, female people, *Dalit* ethnic group, lower BMI people and having lower daily calcium intake. On the other side, alcohol consumption showed the protective effect for osteoporosis. Similarly, educational status, religion, occupation and smoking habit have no effect on the occurrence of osteoporosis in the *Madhesh* province of Nepal.

### Prevalence of osteoporosis

The prevalence of osteoporosis in people aged 50 years and older was 22.3%, which is similar to the findings of other studies [26, 28] but higher than the prevalence of 14.3% found in the study of Yang et al., [50]. This difference might be because later studies were performed in developed countries, where dietary intake can be assumed to be better than that in developing countries. However, a higher prevalence of osteoporosis (34.6 to 37.3%) was also found with DXA [20, 51]. This is due to the difference in the modality of assessing osteoporosis, which can be justified from this study [52]. DXA is considered the gold standard method for bone mineral density measurement [21, 53]. Even though the OSTA has good sensitivity, its specificity compared to that of DXA is moderate [28]. However, the NRS-2002 can be used as a reliable screening tool for osteoporosis in the community setting, as it has shown a sensitivity of 88% for osteoporosis of the femoral neck [54]. On the other hand, the DXA test is relatively expensive and needs to be performed at referral centres [55]; therefore, it is infrequently used in developing countries due to the lack of available equipment [29]. The OSTA is the most practical and accurate tool for daily use in assessing the risk of osteoporosis in the femoral neck [56] because it is a simple, non-invasive, inexpensive and ionizing radiation-free osteoporosis risk screening tool [49]. The cut-off point of -1 for postmenopausal women and men (50–90 years old) yielded a sensitivity of 91% and 81%, respectively, compared with that of DXA [28]. In addition, our findings can be considered reliable because most of the participants in our study were older, and the study indicates that sensitivity of the OSTA increases with age [50].

### Associations of osteoporosis with different factors

#### Osteoporosis and sociodemographic factors

This study revealed that age and sex are positively associated with osteoporosis, which is not surprising. The combination of genetic, hormonal, biochemical, and environmental factors leads to gradual bone loss with age [57]. Females have a greater incidence of osteoporosis than males because females start losing bone at an earlier age and at a faster rate than men [58]. This is due to differences in hormonal effects between the two sexes, such as a lack of estrogen in women after menopause. Additionally, women with a higher incidence of hyperparathyroidism tend to live significantly longer than men with a sixfold greater incidence of osteoporosis in females than in males due to the greater incidence of advanced age [59]. Similarly, the greater bone density in males is also due to the exclusively larger size of the bone in males [60]. Accordingly, female show reduced cortical area (6–25%) than expected for their body size and bone size, which in part signifies the reduced bone strength, when compared with more robust bone of male [61].

In our study, the bivariate analysis revealed that as education level increased, the incidence of osteoporosis decreased, which was supported by the findings of the study of Etemadifar et al. 2013; however, they stated that high literacy is not associated with osteoporosis prevention-related life habits [62]. Another systematic review and meta-analysis revealed that providing education about osteoporosis to adolescents improved their preventive behaviours [63]. Different randomized controlled trials based on health education and management for osteoporosis reveal the better outcome in the term of pain, quality of life, bone mineral density, physical activity, physical strength, health beliefs, living behaviours and others [64, 65]. Similarly, through bivariate analysis, this study revealed that ethnicity affects the occurrence of osteoporosis, which is consistent with the findings of previous study [66]. Variations in body size and composition occur among different ethnic groups and are likely to contribute to osteoporosis [67]. Most of the participants in our study were *Tharus* (55.9%), the oldest and largest indigenous tribe of Nepal, who have their own type of cultural food practice [39]. A study revealed that the incidence of malaria in *Tharus* was nearly seven times lower than that in sympatric non-*Tharus* species [68]. It is assumed that the consumption of freshwater snails plays a vital role in preventing malaria, as snails have a sufficient amount of iron (100 g of edible snail gives 100.7 mg iron) [69], preventing anaemia, and anaemia is positively associated with malaria [70]. Furthermore, a high level of hemoglobin has a protective effect on osteoporosis [71]. On the other hand, the 2016 Nepal Demographic Health Survey (NDHS) revealed that 76% of *Dalit* women experience food insecurity [72], which is positively associated with osteoporosis [73]. However, multivariate analysis did not reveal any association between osteoporosis and ethnicity. Similarly, religion and occupation were not associated with osteoporosis in any of the participants in this study. This might be the reason that most of the participants were farmers (98.2%) and Hindus (93.2%).

#### Osteoporosis and calcium intake

Our study revealed that dietary calcium consumption is negatively associated with osteoporosis. High dietary calcium intake prevents people from developing osteoporosis [74, 75]. This can be justified by the fact that it helps to reduce the enlargement of the appendicular bones, which occurs due to the aging process, and slows bone turnover, reducing the number of active bone remodelling sites [76]. Similarly, calcium acts as a weak antiresorptive agent by reducing bone turnover by approximately 20% through the suppression of parathyroid hormone secretion [77]. Parathyroid hormone reduces the concentration of calcium and stimulates both osteoclast-mediated bone resorption and osteoblast-mediated bone formation, increasing bone turnover [78, 79]. Furthermore, patients with inadequate calcium consumption develop secondary hyperparathyroidism, which leads to increased bone turnover and osteoporosis [80]. However, our multivariate analysis revealed that sufficient calcium-rich food intake was not associated with osteoporosis. One study revealed that an increase in calcium intake among most older adults is unlikely to have any benefit in terms of a reduction in bone loss [81]. In our study, more than half of the participants were from higher age groups (60 years and above).

#### Osteoporosis and BMI

In our study, underweight people were more likely to have osteoporosis than individuals who had a normal BMI or who were overweight/obese. This finding is consistent with different studies [82–84]. It is hypothesized that underweight in human being is frequently related to malnutrition which leads to bone deterioration and osteoporosis [85]. In vitamin D and protein deficiency; vitamin D deficiency impairs the mineralization of the collagenous matrix (colloids), and low protein intake affects bone remodelling by reducing the production of insulin-like growth factor [86]. On the other hand, studies have revealed that anthropometric measurements, such as high BMI, are protective against osteoporosis, and a high waist-to-hip ratio (WHR) is a risk factor for osteoporosis [87, 88]. Individuals with a high BMI experience a large mechanical load on the bone, which increases the bone mass to accommodate this load, hence preventing osteoporosis [89]. It has been found that the increment of one unit in BMI leads to an increase of 0.0082 g/cm^2^ BMD in US older adults [90].

#### Osteoporosis and lifestyle behaviour

Although smoking is closely associated with osteoporosis and decreased bone mineral density [91], our study revealed no association between osteoporosis and smoking, which was also explored in one of the systematic reviews [92]. This might be because, in our study, less than half (44.6%) were female. Ward & Klesges [92] mention that smoking influences reproductive hormones in females, causing natural menopause an average of 1–2 years earlier; hence, females develop osteoporosis, which is not common among males, and furthermore, the effect of smoking on bone is cumulative and dose dependent [92]. Similarly, other confounding variables might have contributed to the lack of a significant association. Additionally, we did not collect data regarding the type or status of smoking, whether it was a past smoker or current smoker.

Bivariate analysis revealed that alcohol consumption has a protective effect on osteoporosis, which is supported by the interventional study of Trius-Soler et al., 2022 that reveals the increment of different bone formation markers with the consumption of alcohol [93]. The effect of alcohol consumption is dose dependent, and a moderate level of alcohol consumption is associated with a greater BMD than heavy alcohol consumption and nondrinker consumption, but a high dose of alcohol leads to osteoporotic fracture [94, 95]. Chronic alcoholism results in a disturbed level of vitamin D metabolism and the development of a low level of serum 25-hydroxyvitamin D [25(OH)D], ultimately leading to osteoporosis [96]. However, the multivariate analysis did not show any association of osteoporosis with alcohol consumption in our study, which is consistent with the study of Khiyali et al. [97].

## Limitations of the study

Laboratory parameters and radiological findings were not used in this study, the findings cannot be applied in the clinical setting. Similarly, recall bias may have occurred. Our effort was focused on minimizing these types of recall errors by increasing the amount of time participants spent. Additionally, we could not explore the socioeconomic status of the participants, as a high incidence of osteoporosis is associated with a low socioeconomic status [98]. Similarly, we did not use software to calculate the amount of calcium in specific foods. Instead, we manually calculated the portion sizes of food consumed by participants, which may have resulted in errors in estimating food intake. Besides these, this study has several strengths. There are limited studies on the prevalence of osteoporosis using the OSTA score. Moreover, no studies have explored the association between osteoporosis and calcium-rich food consumption. This research may be the first of its kind in this area. It could shed light other researchers to conduct further studies on osteoporosis from a nutritional perspective, an area that is currently lacking.

## Conclusions

This study revealed a high prevalence of osteoporosis among individuals aged 50 years and older in the *Madhesh* Province of Nepal due to the combined effects of low BMI (underweight) and inadequate daily calcium intake. Nutritional counselling services might encourage people to consume sufficient calcium-rich food and adopt appropriate lifestyle behaviours to maintain healthy body weight so that osteoporosis and osteoporotic fractures can be prevented. Further research can explore the impact of socioeconomic status and medical comorbidities on a large scale.

## Data Availability

No datasets were generated or analysed during the current study.

## Abbreviations

BMD: Bone mineral density
BMI: Body mass index
DXA: Dual-energy X-ray absorptiometry
ERB: Ethical Review Board
IQR: Interquartile range
NHRC: Nepal Health Research Council
OSTA: Osteoporosis Self-assessment tools for Asians
